# Segmentation and Clustering Analysis of Scanned Foot Models for Intelligent Diabetic Foot Ulcer Offloading Boots

**DOI:** 10.1007/s10278-025-01746-6

**Published:** 2025-11-10

**Authors:** Yaser Sabzehmeidani, Andrei-Alexandru Popa

**Affiliations:** The Faculty of Engineering, Institute of Mechanical and Electrical Engineering, SDU Mechatronics (CIM), University of Southern Denmark (SDU), Alsion 2, 6400 Sønderborg, Denmark

**Keywords:** Diabetic foot ulcer (DFU), 3D foot scanning, Clustering algorithms, Segmentation, Rehabilitation boot

## Abstract

Diabetic foot ulcer (DFU) is a common and severe complication of diabetes that leads to amputation if not effectively managed. Intelligent offloading or rehabilitation devices or boots are used to manage or observe the ulcer and the process of treatment which requires the segmentation and clustering analysis of 3D scanned foot models. This study examines the effectiveness of two widely used clustering algorithms, K-Means and Gaussian Mixture Models (GMM), in segmenting scanned foot models to use in rehabilitation boots. The performance of K-Means and GMM was compared across 98 foot models. GMM consistently achieved higher silhouette scores (0.58 vs. 0.42 for *K* = 5), lower Davies-Bouldin scores (0.47 vs. 0.54 for *K* = 5), and more stable clustering across anatomical sections, despite requiring almost 20% more computation time. These results highlight GMM’s superior ability to capture the complex nonlinear structures of diabetic feet, with implications for more precise and personalized offloading boot design. Precise segmentation of scanned foot models is a crucial step in various anatomical and medical applications, such as the design of custom orthotic devices, rehabilitation offloading boots, and the analysis of foot biomechanics—particularly useful within DFU management.

## Introduction

Diabetic foot ulcer (DFU) and the complications happening for the patients like amputation are a significant concern for diabetic patients, leading to severe health implications and reduced quality of life. Current clinical solutions to DFU treatment rely heavily on patient and clinician care and have significant limitations like high costs and lengthy care [1]. To overcome these challenges, medical researchers have explored the use of intelligent boot casts to assist in offloading and rehabilitation. Recent advancements in artificial intelligence and machine learning have enabled novel approaches to personalized offloading device prescriptions, leveraging the analysis of 3D scanned foot models. Segmentation and clustering of 3D scanned foot models play a critical role in identifying high-risk plantar regions that are prone to ulceration and help in the offloading solution for rehabilitation purposes. Clustering outcomes, such as silhouette scores and Davies-Bouldin Index (DBI) values, provide an objective measure of how accurately anatomical regions are delineated. Higher clustering validity directly translates into more reliable identification of pressure zones (e.g., forefoot, midfoot, heel), which can be incorporated into personalized offloading boot design.

The standard method starts with applying the segmentation process to a dataset of collected 3D-scanned diabetic feet. Each segmented region is then subjected to clustering analysis to identify patterns and similarities, as the insights gained from this analysis will directly inform the design, lattice structure, and manufacturing process of an intelligent offloading boot tailored for DFU patients. By leveraging these data-driven approaches, the final product aims to provide improved fit, targeted pressure relief, and enhanced comfort for patients at risk of foot ulcers. Accurate segmentation ensures that offloading structures align with vulnerable regions of the foot, thereby reducing peak plantar pressures, preventing ulcer recurrence, and improving patient adherence. This clinical linkage underscores the importance of evaluating clustering methods not only for computational performance but also for their potential impact on DFU management.

Cluster analysis is a data mining technique that groups similar data points into clusters based on their fundamental characteristics [2]. It could be an unsupervised machine learning solution that organizes and classifies objects or data points into different clusters according to similarities or patterns. Image segmentation has widely been adopted by clustering to separate images into meaningful groups or objects [2, 3]. For the design of custom orthotics and the analysis of foot biomechanics, reliable segmentation of scanned foot models is vital. Particularly for DFU patients with ulcers in their foot or toes, custom wearables must be implemented for rehabilitation and offloading of plantar stress. The accurate segmentation of scanned foot models is a crucial step in the development of personalized orthotic devices, as it enables the identification of key anatomical features that can be used to inform the design process. To allow this, foot scanning and regenerating mesh models out of the scanned data are necessary steps. In the context of image segmentation, two of the most used clustering algorithms are K-Means and GMM [4–6]. The K-Means method is a relatively simple and efficient algorithm that splits the data into *k* clusters based on the similarity of data points to the cluster centroids. This rugged approach is, however, linked to sensitivity to initial conditions and the inability to capture non-convex or stretched clusters. GMM on the other hand is a more flexible probabilistic approach that can model complex cluster shapes by assuming that the data is generated from a mixture of Gaussian distributions. Despite the widespread use of these clustering algorithms in image segmentation, there is limited research on their performance for the specific task of scanned foot model segmentation [7]. The current effort aims to address this research gap, with the goal of informing the choice of and benchmarking algorithms for healthcare professionals working with, among others, DFU management.

The results of this study have significant implications for the design and fabrication of custom ankle–foot orthoses (AFO), Total Contact Cast (TCC), or specialized therapeutic footwears [8–10]. Previous research has explored the use of 3D scanning and printing techniques for the manufacture of custom-made foot orthoses [11, 12]. By precisely capturing the complex geometry of the foot, the segmentation approach can facilitate the development of customized orthotic devices that better align with the individual’s unique foot shape and biomechanics [10, 13–15]. This improvement in the design process can lead to enhanced comfort, better functionality, and overall effectiveness of the orthotic solutions, ultimately benefiting patients with various foot-related conditions [13, 16–19].

## Methods

**Fig. 1:**
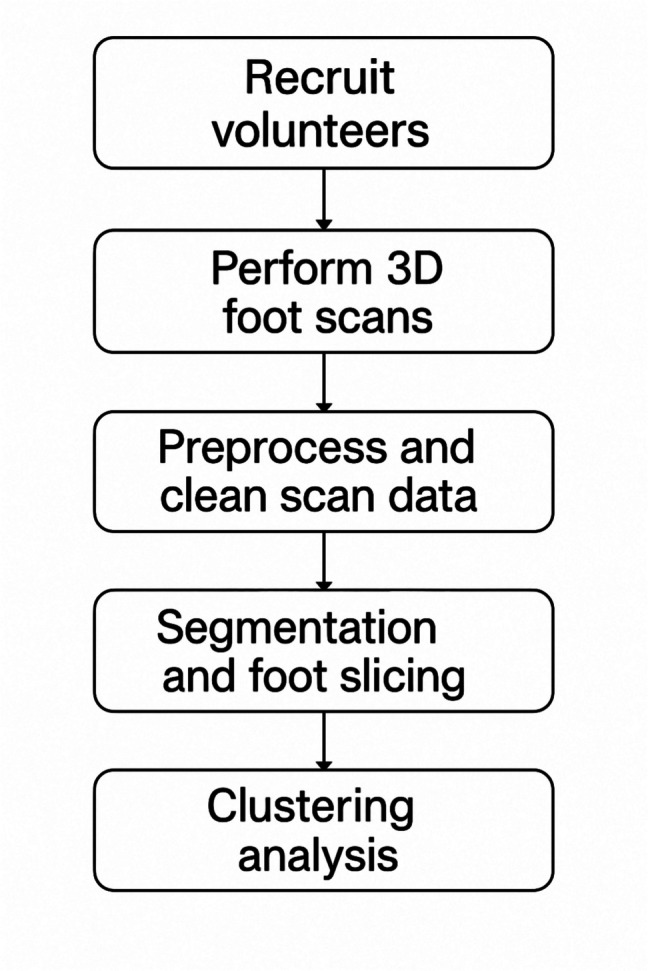
Flowchart of scanning and clustering workflow

This study was approved by the Research Ethics Committee of the Southern University of Denmark (Approval No: 12.342). Written informed consent was obtained from all participants prior to scanning. A structured scanning procedure was implemented as part of the overall data acquisition methodology. The process began with the recruitment of volunteers representing a wide range of foot sizes and anatomical profiles to capture sufficient variation within the dataset. Following this, 3D foot scans were performed, capturing detailed geometrical features of both the plantar and dorsal surfaces of foot. The raw scan data were then preprocessed to remove noise and artifacts, followed by mesh alignment and smoothing to ensure data quality. Preprocessing involved three stages: noise removal, mesh alignment, and outlier handling. Noise was reduced using a statistical outlier filter with a threshold of two standard deviations, eliminating approximately 3% of points per scan. Mesh alignment was performed using dedicated software to achieve a mean root-mean-square alignment error of 1.5 mm across all scans. Outliers were excluded by applying a three-time distance threshold relative to local neighborhood points, which reduced point sets by less than 5%. After preprocessing, the average point cloud density was 1.2 × 10^5^ points/cm^2^, confirming that the meshes retained sufficient resolution for clustering analysis. Next, relevant anatomical sections were considered for segmentation to support further analysis. Finally, the cleaned and annotated datasets were securely stored for subsequent applications such as clustering, statistical analysis, and model development. The overall scanning and clustering workflow is illustrated in Fig. 1.

Clustering analysis is a commonly used technique for segmenting human-related scanned models [20, 21]. To evaluate the performance of these two considered methods, a dataset of 98 scanned foot models was collected at the Southern University of Denmark, at the Sønderborg campus. The scanned models were manually segmented into ten different slices to regenerate the median model for subsequent analysis. The number of clusters (*n* = 3, 5, and 7) was selected to reflect potential offloading boot sizing strategies for the target population. For example, *n* = 3 corresponds to three general boot sizes (small, medium, large), *n* = 5 represents a more granular size distribution (very small, small, medium, large, very large), and *n* = 7 allows for even finer gradations across the population. This choice ensures that the clustering outcomes directly align with practical manufacturing and clinical application needs. K-Means is computationally more efficient than GMM, especially for large datasets, because it involves simple distance calculations and centroid updates. In contrast, GMM requires estimating multiple parameters for each Gaussian component, such as mean, variance, and mixing coefficients, which can be computationally expensive and sensitive to initialization [22, 23]. The K-Means and GMM algorithms were both applied to the dataset, and the resulting segmentations were compared. The flexibility of GMM in modeling complex data distributions can be advantageous for segmenting scanned foot models, as it can better capture the gradual transitions and overlapping regions between different anatomical structures [24, 25]. This flexible modeling technique allows GMM to capture more complex cluster shapes and nonlinear data structures compared to the simpler K-Means clustering algorithm. Unlike K-Means, which uses hard assignments, GMM employs soft assignments, where each point belongs to all clusters with varying probabilities. This flexibility allows GMM to model more complex cluster shapes, such as ellipsoidal or overlapping clusters, making it more suitable for datasets with non-uniform geometries, such as scanned foot models. The underlying mathematics of both algorithms is described in the next section, followed by the data acquisition description.

### K-Means

Widely used as a clustering algorithm due to its relative simplicity, K-means aims to minimize the within-cluster variance by the number of clusters defined as k [20]. It is a centroid-based clustering method that assumes clusters are spherical and minimizes the within-cluster variance. To do so, the algorithm begins by randomly selecting k points as the initial cluster centroids to minimize the Sum of Squared Distance (SSD) between data points and respective cluster centroids as below.

1 $$J=\sum\nolimits_{i=1}^{k}\sum\nolimits_{x\in {C}_{i}}{\Vert x-{\mu }_{i}\Vert }^{2}$$

Here, J is the objective function, k is the number of clusters, x is the data point, ${C}_{i}$ is the cluster i, and ${\mu }_{i}$ is the centroid of cluster i. Each data point is then assigned to the nearest centroid, and the centroids are updated to be the mean of the assigned data points. The centroid of each cluster is updated as the mean of all points as:

2 $${\mu }_{i}=\frac{1}{\left|{C}_{i}\right|}\sum\nolimits_{x\in {C}_{i}}x$$

The variable ${\mu }_{i}$ depicts the updated centroid of cluster i and $\left|{C}_{i}\right|$ is the number of points in cluster ${C}_{i}$. It partitions the data into k clusters by iteratively updating cluster centroids and assigning points to the nearest centroid. This simplicity makes it computationally efficient, but it struggles with non-spherical or overlapping clusters and requires the user to specify the number of clusters beforehand. The distance metric in the K-Means is measured between data point x and a centroid ${\mu }_{i}$.

3 $$\Vert x-{\mu }_{i}\Vert =\sqrt{\sum\nolimits_{j=1}^{d}{({x}_{j}-{\mu }_{i,j})}^{2}}$$

The dimensionality of the data is denoted by *d*, ${x}_{j}$ is the j-th feature of the data point x, and ${\mu }_{i,j}$ is the j-th feature of the centroid ${\mu }_{i}$. In practice, K-Means is preferred when speed and simplicity are prioritized, and the dataset’s structure roughly matches its assumptions. K-Means is a popular choice for image segmentation tasks due to its simplicity and efficiency, but it may not be well-suited for complex data structures with non-convex or overlapping clusters. The algorithm’s limitations can be mitigated by careful parameter selection, preprocessing steps, and the use of more advanced clustering techniques, such as Gaussian Mixture Models, which can better handle complex data distributions [5, 26].

### Gaussian Mixture Models (Gmms)

GMMs employ a probabilistic approach to represent the data as a composite of Gaussian distributions. This model depicts the data as a weighted combination of Gaussian components, with each component denoting a distinct cluster [7]. In the GMM method, the Gaussian probability density function is defined as:

4 $$N\left(x|{\mu }_{k},\sum k\right)=\frac{1}{{(2\pi )}^{d/2}{\left|\sum k\right|}^{1/2 }}\mathrm{exp}(-\frac{1}{2}{(x-{\mu }_{k})}^{T}{\sum }_{k}^{-1}(x-{\mu }_{k}))$$

The variable ${\mu }_{k}$ is the mean of the k-th Gaussian component, ∑k is the covariance of k-th Gaussian component, d is the dimensionality of the data, and $\left|\sum k\right|$ is the determinant of the coverage. The Expectation–Maximization algorithm is utilized to learn the parameters of the Gaussian components, including the means, covariances, and mixing weights. For weighted probability, it would derive as below:

5 $${\gamma }_{{z}_{ik}}=\frac{{\pi }_{k}N\left({x}_{i}|{\mu }_{k},\sum k\right)}{{\sum }_{j=1}^{K}{\pi }_{j}N\left({x}_{i}|{\mu }_{j},\sum j\right)}$$

The ${\pi }_{k}$, K is the number of gaussian components and ${\gamma }_{{z}_{ik}}$ is the responsibility of gaussian k for the data point ${x}_{i}$. The optimal number of Gaussian components is determined through an iterative optimization process that aims to maximize the likelihood of the data given the model parameters. The GMM algorithm iteratively estimates the optimal model parameters, such as the mean, covariance, and mixing proportions of the Gaussian components, to maximize the likelihood of the observed data. The updated mixing coefficient is taken from ${\pi }_{k}=\frac{1}{N}{\sum }_{i=1}^{N}{\gamma }_{{z}_{ik}}$ while the means is extracted from ${\mu }_{k}=\frac{{\sum }_{i=1}^{N}{\gamma }_{{z}_{ik}}{x}_{i}}{{\sum }_{i=1}^{N}{\gamma }_{{z}_{ik}}}$.

The flexibility of GMM in modeling complex data distributions can be advantageous for segmenting scanned foot models, as it can better capture the gradual transitions and overlapping regions between different anatomical structures. This flexible modeling technique allows GMM to capture more complex cluster shapes and nonlinear data structures compared to the simpler K-Means clustering algorithm [27]. As stated previously, K-Means uses hard assignments while GMM employs soft assignments, where each point belongs to all clusters with varying probabilities. This flexibility allows GMM to model more complex cluster shapes, such as ellipsoidal or overlapping clusters, making it more suitable for datasets with non-uniform geometries, such as scanned foot models.

**Fig. 2:**
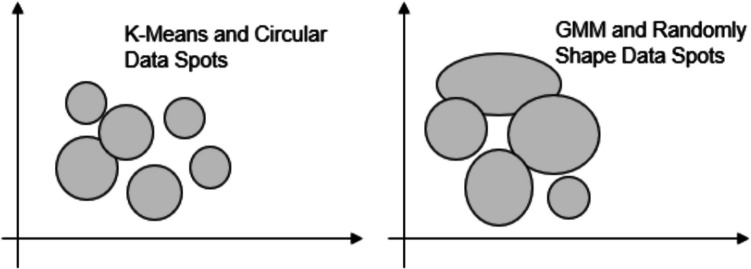
A comparison of K-Means and GMM methods

Both methods benefit from preprocessing steps like dimensionality reduction to improve clustering quality and computational efficiency [28, 29]. For 3D scanned foot models, K-Means may perform well when the data points form compact, spherical clusters, such as clear separations between toes or heel regions. However, it may misclassify regions where shapes overlap or have gradual transitions, such as the arch of the foot. GMM’s ability to model data with overlapping clusters or gradual changes gives it an advantage in these scenarios. For instance, it can better handle the blending of features in complex foot geometries, such as the curvature of the plantar surface (Fig. 2) [30–33].

### Scans Setup and Preprocessing

**Fig. 3:**
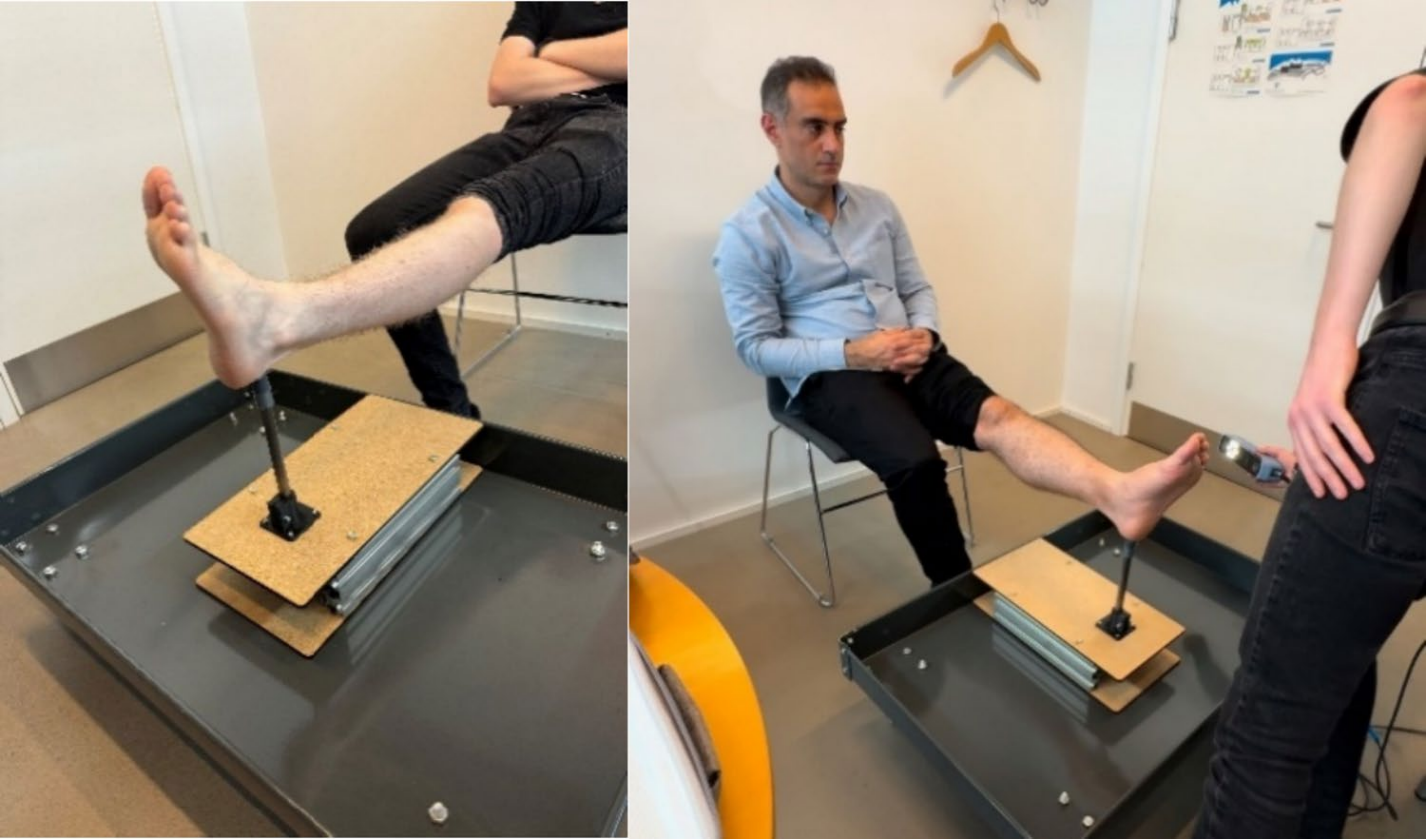
Setup and preparation (left) and scan in progress (right, image reproduced with written consent from participant)

**Fig. 4:**
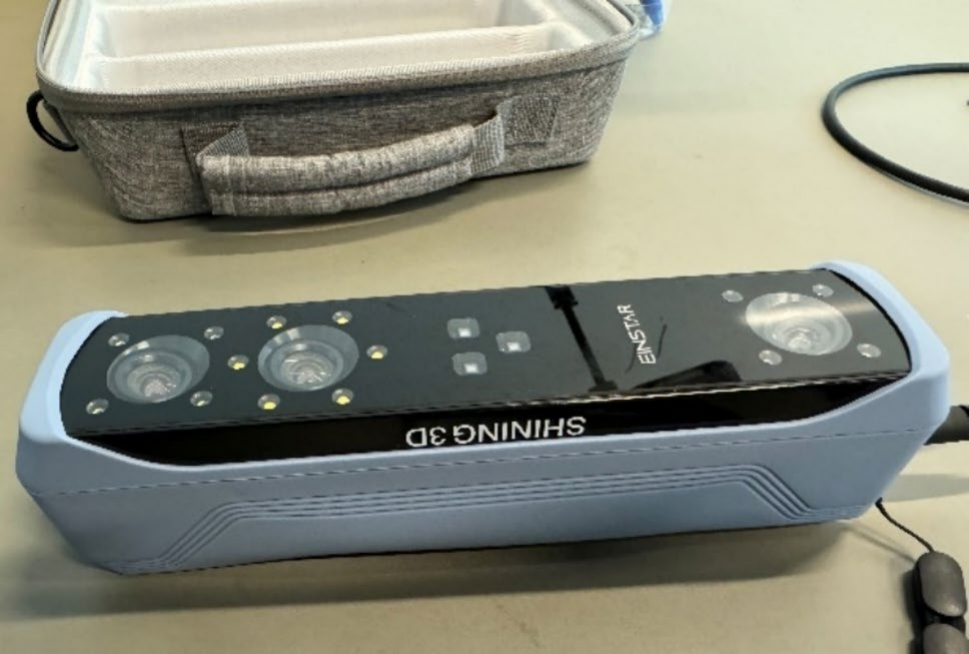
Shining 3 d (Einstar) portable 3D scanner

**Fig. 5:**
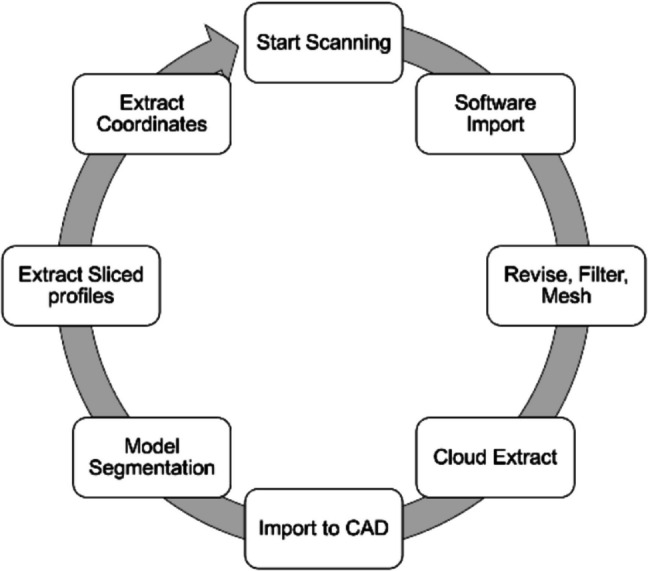
Data capturing procedure flowchart for each volunteer

The foot models were obtained using a 3D scanning system and then preprocessing and postprocessing steps to remove extra scanned parts, noise, and outliers. A 3D photo laser scanner was employed to capture precise surface geometry and texture details. A portable 3D scanner from Shining3D, the Einstar, was utilized for data collection. The associated hardware featured dedicated 3D scanning software capable of exporting the scanned data in OBJ/STL format. The scanning process was accurately planned and executed to ensure the acquisition of high-quality data. This advanced technology guaranteed the accuracy and reliability of the data, a critical factor for subsequent analyses and modeling. The data acquisition campaign involved a total of 98 volunteer participants, resulting in a diverse dataset for analysis (Figs. 3, 4, 5).

The Einstar ExStar software was utilized to facilitate the scanning and postprocessing workflow. Initially, the software was employed to capture detailed scans of the subjects, generating high-resolution mesh clouds that accurately represented the scanned geometries. Following the initial capture, the mesh clouds underwent necessary editing to clean up the data, ensuring clarity and eliminating unnecessary noise such as unwanted objects or irrelevant scanned body parts, in order to enhance the quality and suitability of the data. This editing process enabled the extraction of OBJ files, which serve as a standardized format for 3D modeling and analysis. The OBJ files were subsequently imported into CAD software for further processing and refinement. Within the CAD environment, the scans were enhanced through mesh insertion and the careful placement of specific geometries to define regions of interest. Additional steps included the removal of any extraneous mesh clouds or unintended scanned features, ensuring that the models were both precise and streamlined for their intended applications.

After the refinement process, the enhanced models were exported as STP files, a widely adopted format for 3D CAD compatibility. This format conversion ensured compatibility with various engineering and manufacturing workflows, facilitating downstream utilization of the data. These preprocessing steps were applied consistently across foot scan datasets to maintain uniformity. However, the current discourse and accompanying visuals concentrate solely on the foot scan models. The analysis pipeline involved preprocessing techniques such as scan alignment, noise reduction, and outlier removal to enhance the quality and suitability of the data for subsequent segmentation analyses.

## Results

**Fig. 6:**
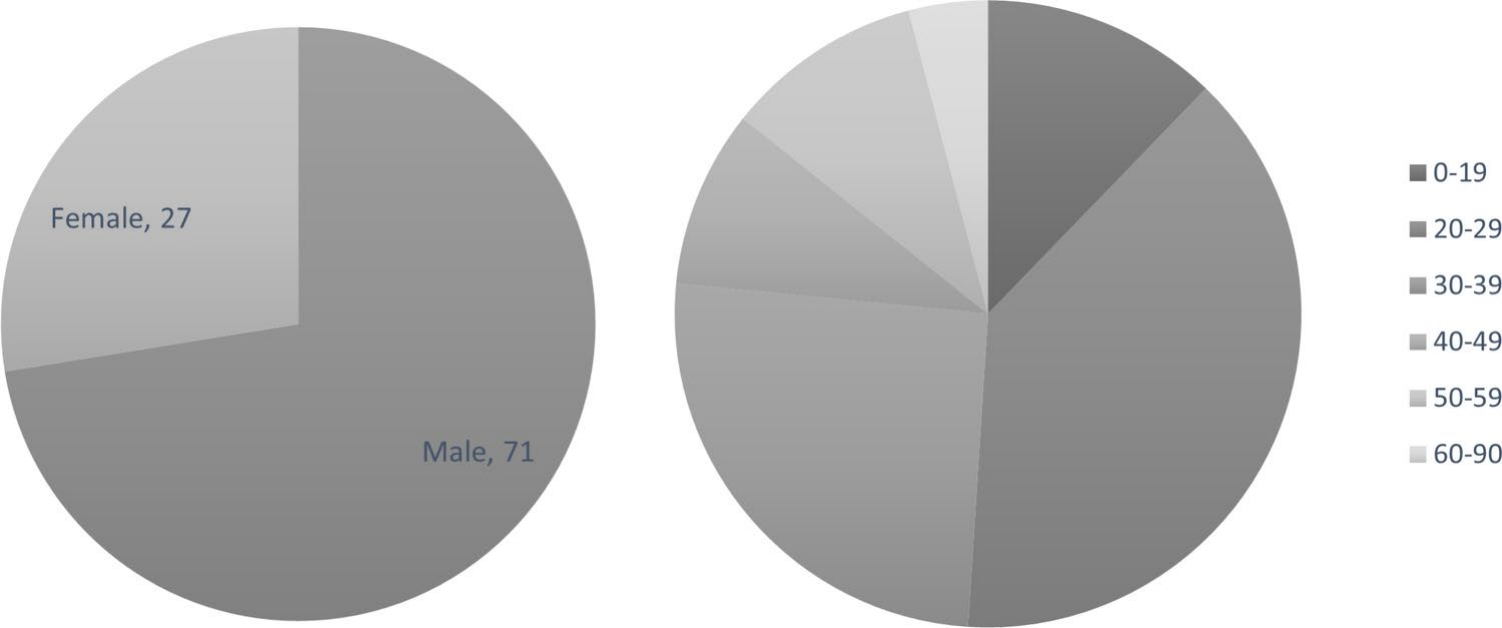
Gender distribution (left) and age distribution (right)

**Fig. 7:**
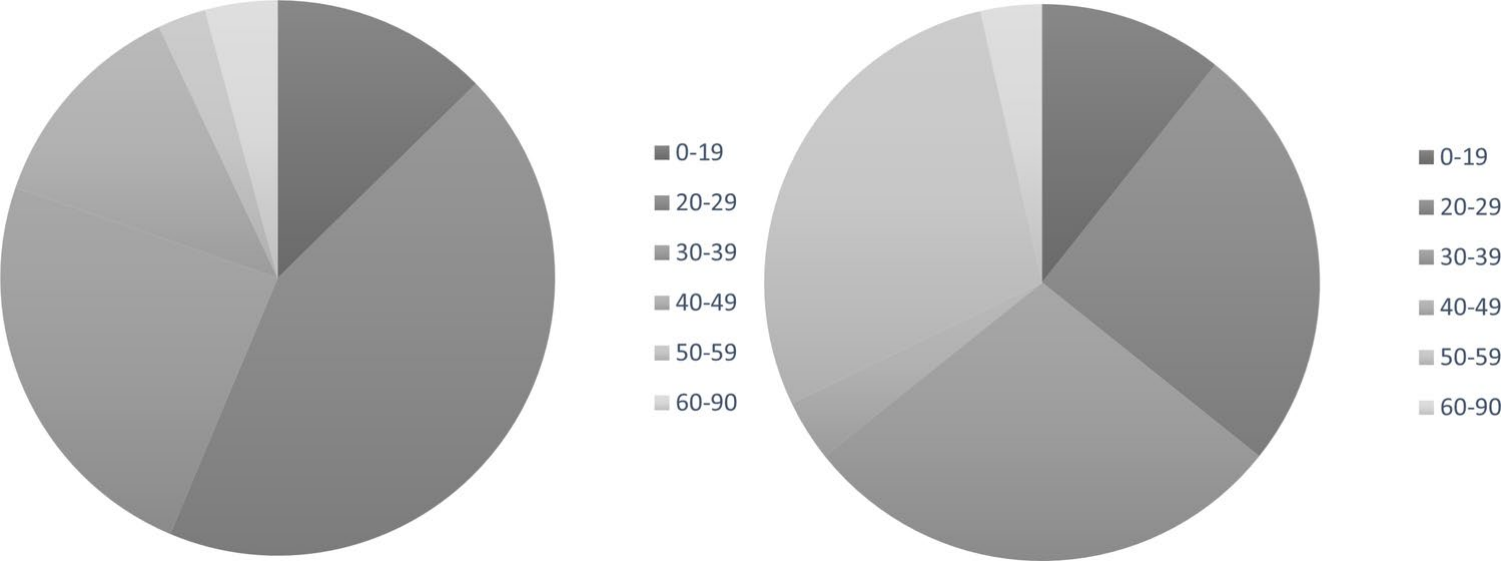
Age distribution, men (left) and women (right)

**Fig. 8:**
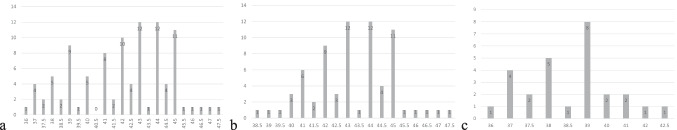
Shoe size distribution and number of participants: **a** all, **b** men, **c** women

The scanning procedure took place at the Southern University of Denmark, Sønderborg campus, with a total of 98 volunteer participants including students and staff. The study participants were selected to encompass a diverse range of demographic characteristics, including variations in gender, age, and body size. The gender distribution, age statistics, and shoe size data of the participants are summarized and visualized in the following graphs (Figs. 6, 7, 8), providing an overview of the dataset’s diversity and representativeness. These insights are crucial for understanding the variability within the target population and tailoring the project’s outcomes accordingly.

**Fig. 9:**
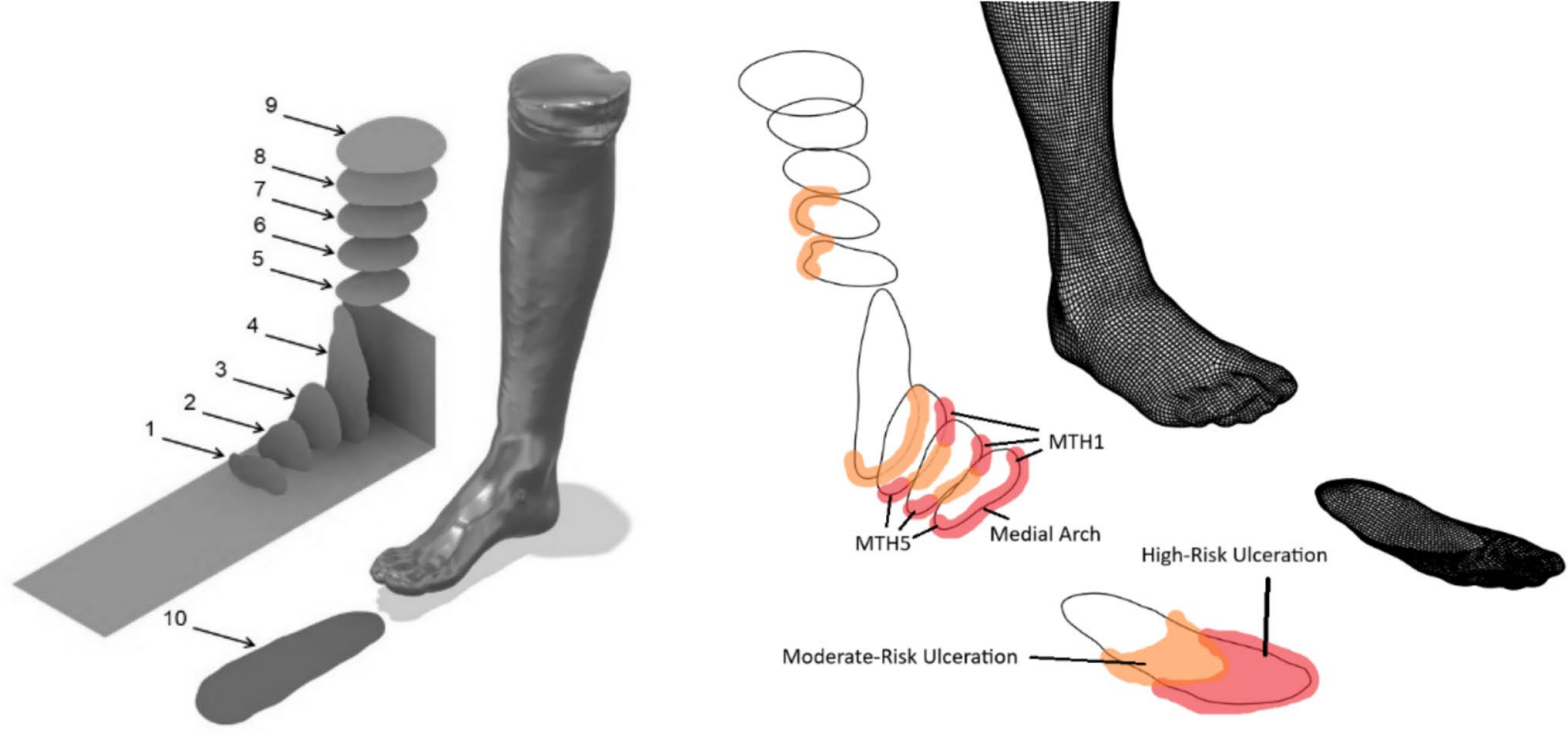
Reference sections defined for analysis (left). Anatomical landmarks and clinically significant plantar regions including MTH (metatarsal head) (right)

After the accomplishment of scans, for the clustering, each foot was sliced into ten sections with reference to foot sole and foot heel anatomy shown in Fig. 9. These defined sections are created with a 40-mm offset from the reference planes, four sections defined for the foot sole, five sections defined for the foot calf, and one-foot sole profile totaling ten reference planes. The 40-mm slicing strategy corresponded to clinically meaningful plantar regions, including forefoot, midfoot, and heel sections, aligning with offloading boot pressure zones. These slices correspond to clinically relevant anatomical regions: section 1–4 represent the forefoot (including metatarsal heads), section 4 overlaps the midfoot arch, section 5–9 cover the calf and ankle regions, and section 10 captures the overall sole profile. This mapping ensures that the segmentation reflects functionally important regions for pressure redistribution and offloading boot design.

**Fig. 10:**
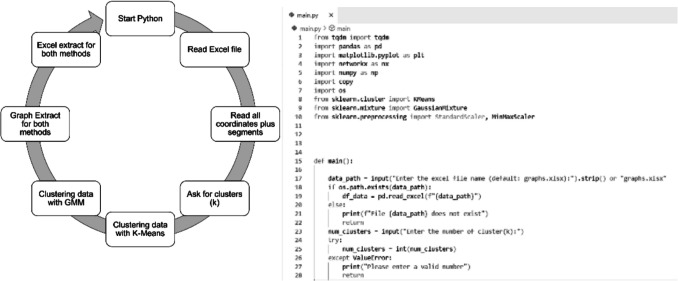
Initials of Python program for data analysis and assessment

To assess and analyze, Python software was used to assist in the segmentation of the processed 3D foot models using the K-Means and GMM algorithms. The program captured all the sections, participants, and coordinates and then extracted and analyzed the profiles of each foot slice based on the algorithms. The K-Means algorithm partitioned the foot model into a predefined number of clusters, while the GMM approach modeled the data using a mixture of Gaussian distributions. The study considered *n* = 3, 5, and 7 clusters to evaluate the performance of these segmentation techniques (Fig. 10).

**Fig. 11:**
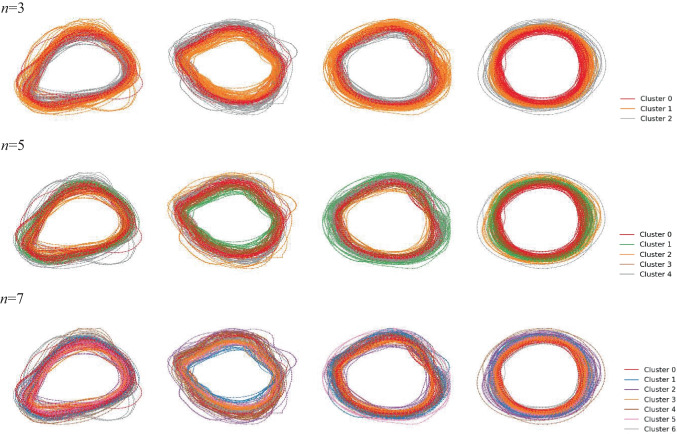
GMM plot of selected sections; section 3, section 5, section 6, section 7 (left to right)

**Fig. 12:**
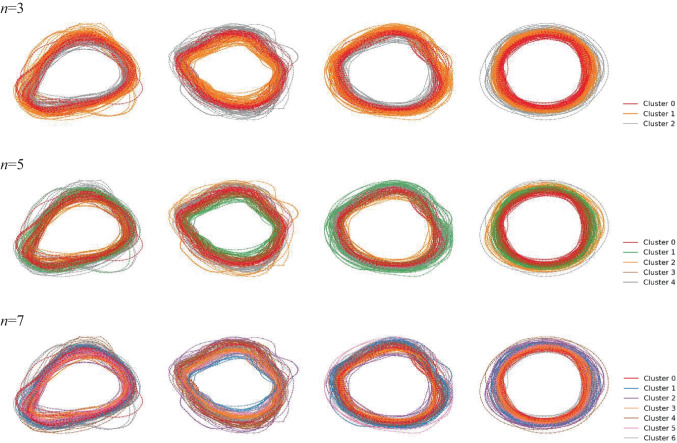
K-Means plot of selected sections; section 3, section 5, section 6, section 7 (left to right)

**Fig. 13:**
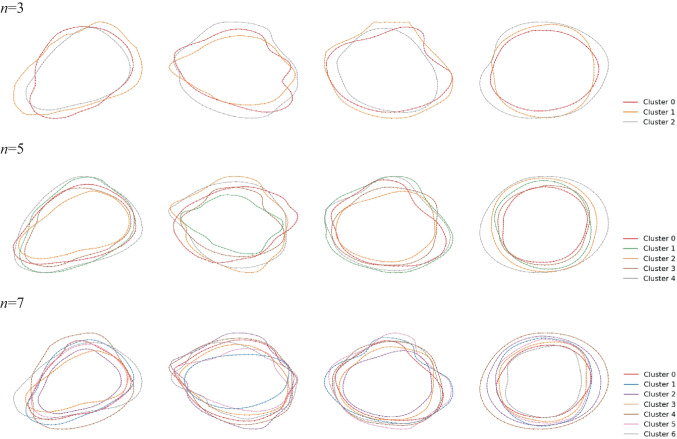
GMM plot of median sections; section 3, section 5, section 6, section 7 (left to right)

**Fig. 14:**
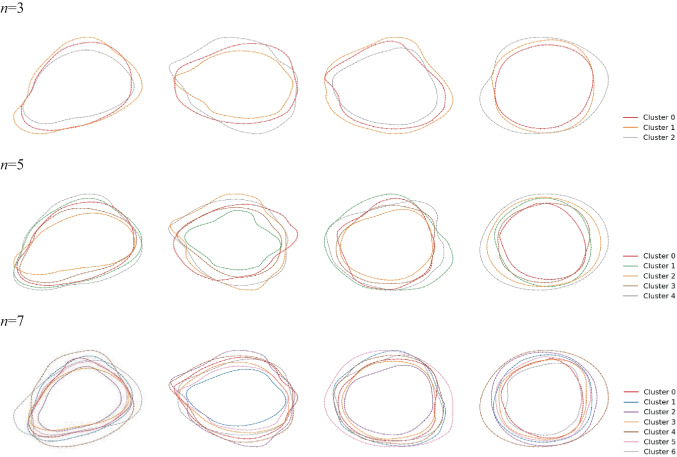
K-Means plot of median sections; section 3, section 5, section 6, section 7 (from left to right)

The foot scan segmentation results using K-Means and GMM algorithms are presented in Figs. 11, 12, 13, and 14, respectively.

## Discussion

**Fig. 15:**
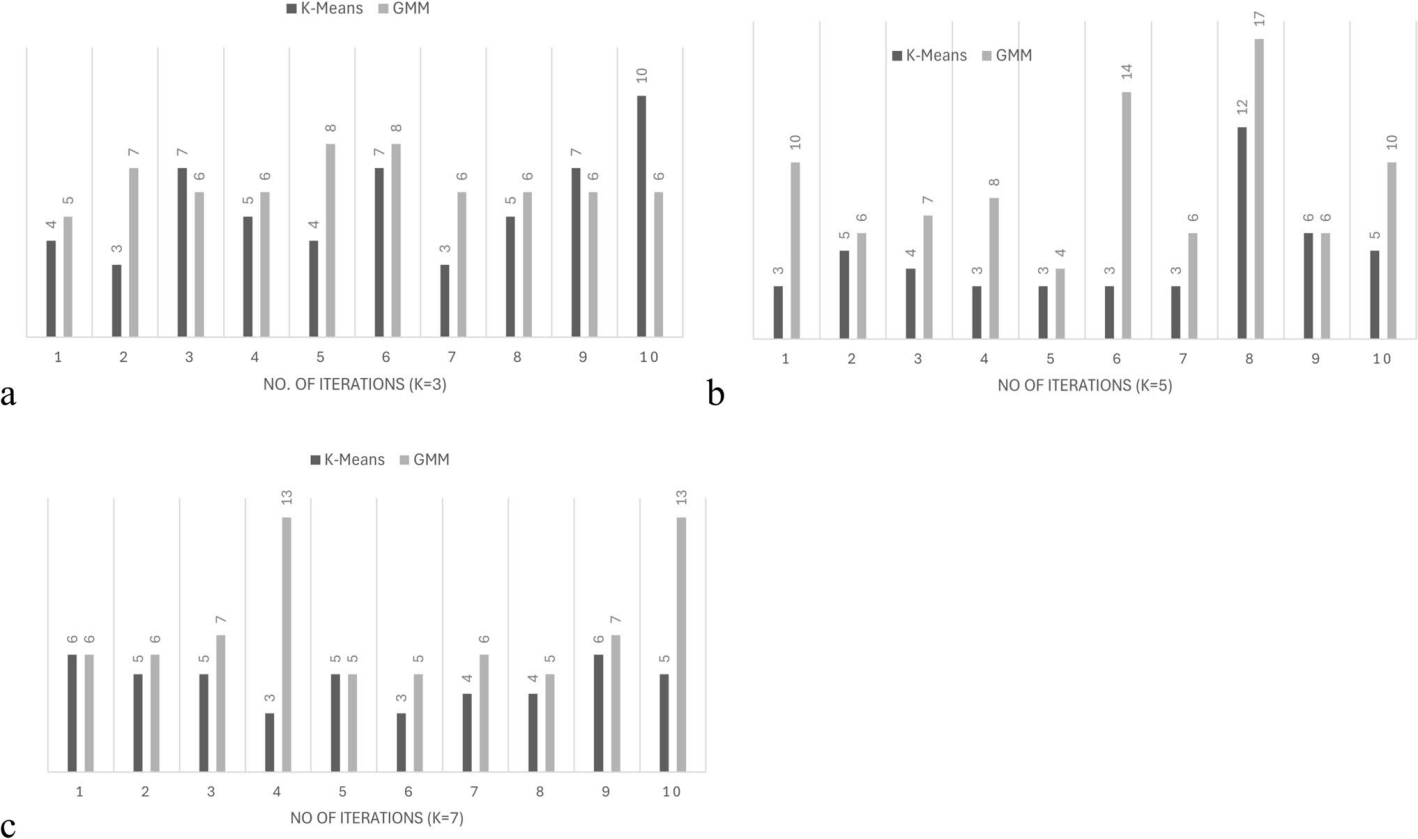
Number of computing iterations for each section: **a ***k* = 3, **b ***k* = 5, **c ***k* = 7

**Fig. 16:**
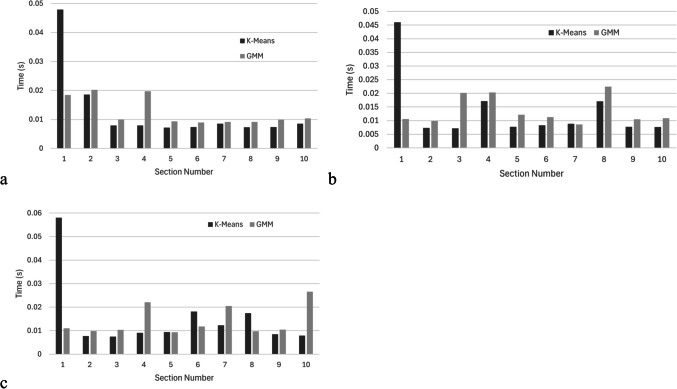
Computing time: **a ***k* = 3, **b ***k* = 5, **c ***k* = 7

**Fig. 17:**
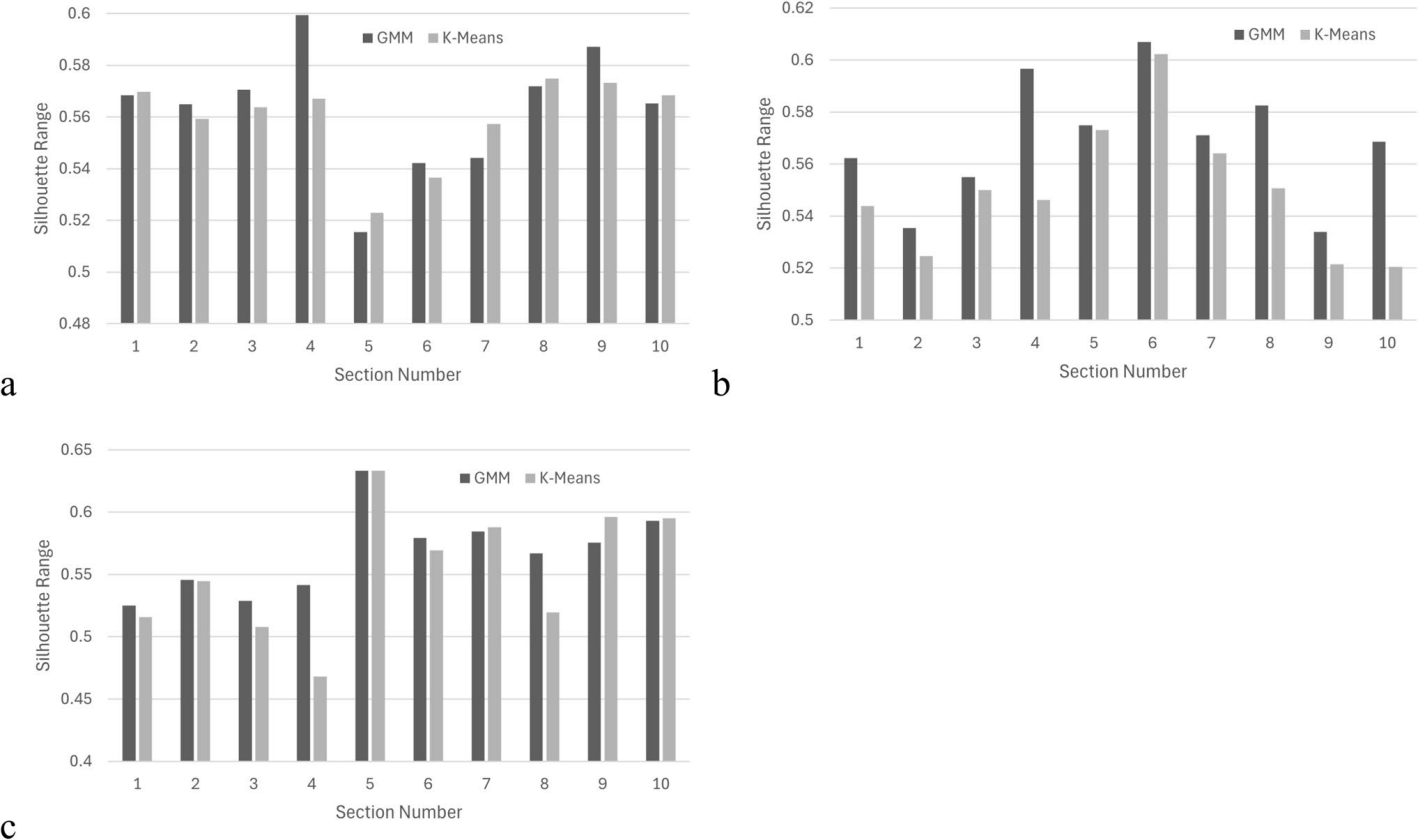
Silhouette score comparison for K-Means and GMM: **a ***k* = 3, **b ***k* = 5, **c ***k* = 7

**Fig. 18:**
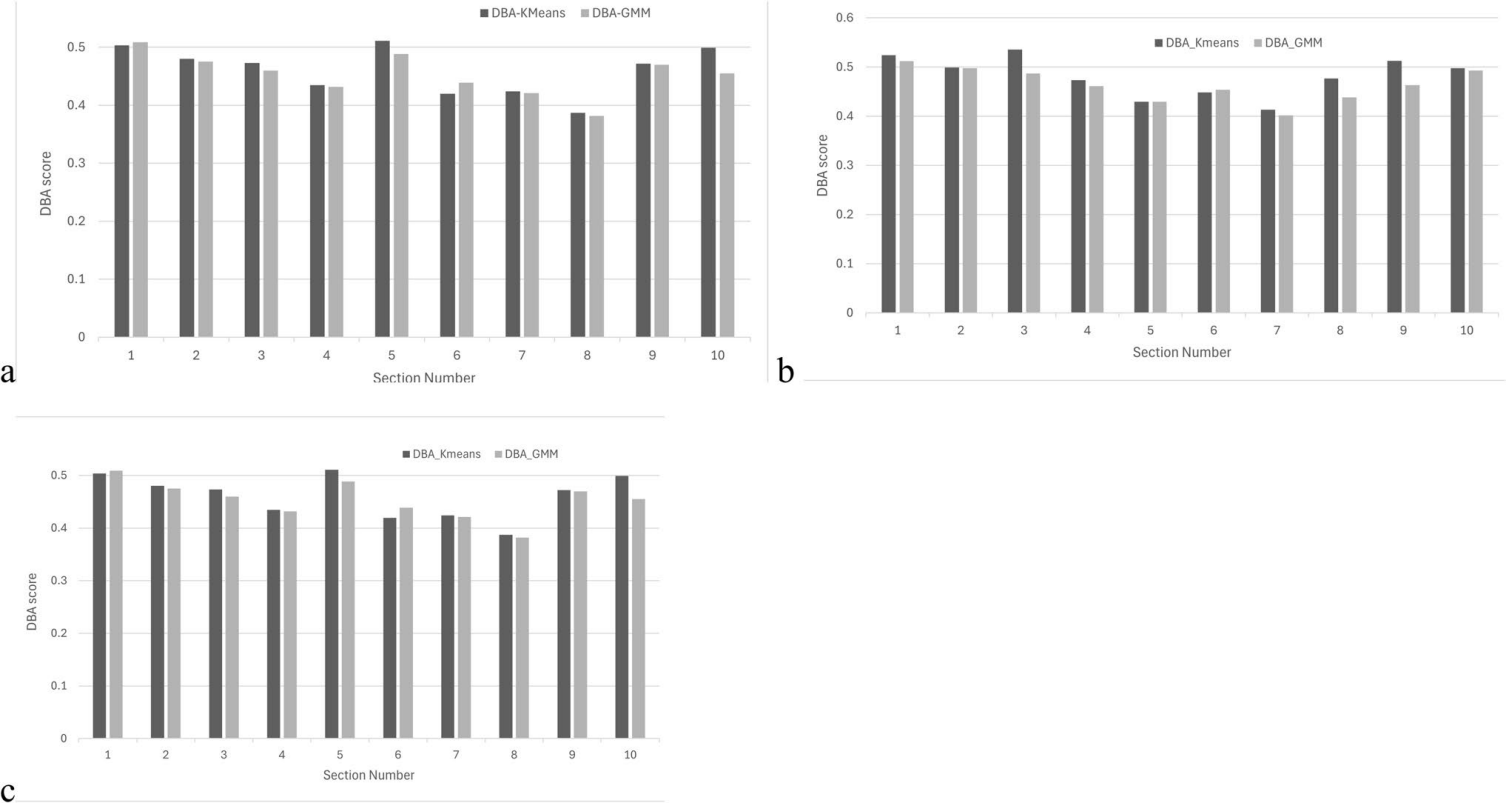
Davies-Bouldin Index (DBI) results for K-Means and GMM: **a ***k* = 3, **b ***k* = 5, **c ***k* = 7

The number of iterations and computation time for each section have been provided below. For the comparison, Silhouette analysis [34] was performed to quantify the quality and cohesion of the clusters generated by the K-Means and GMM approaches. The Silhouette score ranges from − 1 to 1, with higher values indicating better-defined clusters. Alongside silhouette analysis, the Davies-Bouldin Index (DBI) was used to quantify cluster separation and compactness (Figs. 15, 16, 17). Results showed that GMM consistently achieved lower DBI values (mean 0.47 vs. 0.54 for *K* = 5), confirming superior clustering quality compared with K-Means. These results are illustrated in Fig. 18, which summarizes DBI values across cluster size.

Figure 18 shows the DBI results comparing K-Means and GMM for cluster sizes *n* = 3, 5, and 7. Lower DBI values indicate better clustering performance and GMM consistently achieved lower scores.

The investigation compared the performance of the K-Means and GMM techniques in segmenting scanned foot models. Our findings extend prior DFU segmentation studies using convolutional neural networks and foundation models such as MedSAM [7], by demonstrating that classical clustering remains advantageous in computational efficiency and interpretability. Recent advances in foundation models such as MedSAM have achieved superior performance in 3D segmentation. However, these methods require high computational resources and annotated training data, which limit near-term clinical adoption. Classical clustering offers an interpretable, training-free alternative, suitable for integration into CAD/CAM pipelines. Unlike earlier CAD/CAM orthotics studies [11, 12], this work directly links clustering outcomes to offloading boot design parameters, offering a translational bridge between computational analysis and clinical applications. While the K-Means algorithm is simpler and more computationally efficient, it struggles to model the irregular shapes and overlapping clusters observed in the foot scans. On the other hand, the GMM technique, with its capacity to learn the underlying data distribution and assign soft cluster memberships, was able to better represent the intricate structures within the foot models. These results suggest that the GMM approach is a more appropriate choice for the segmentation of intricate 3D foot models, offering a more faithful representation of the foot’s underlying anatomy and biomechanics.

To achieve successful clinical implementation of clustering-driven segmentation, a few factors have to be considered. First, real-time segmentation in clinical environments is critical for adoption. While the current pipeline is computationally feasible, further optimization and software integration are needed to deliver results directly within patient consultation workflows. Second, multi-center validation is necessary to ensure robustness across diverse diabetic populations, as anatomical variation and ulcer risk differ by demographic and geographic factors. Finally, integration with CAD/CAM workflows remains a key step for clinical translation. Clustering outputs must be seamlessly exported into design platforms for orthotic and boot fabrication, which raises challenges related to software interoperability, scan-to-print logistics, and staff training. Addressing these translational issues will be essential to move from proof-of-concept to routine clinical use.

### Limitations

The study population was limited to a Danish cohort and participant students and staff, which may not reflect global DFU demographics or racial anatomical variations. The manual slicing method may not fully align with all clinical anatomical landmarks. Moreover, only classical clustering algorithms were tested; future research should integrate AI-driven segmentation, such as CNNs or foundation models, and validate across multi-center cohorts.

## Conclusion

The segmentation and clustering analysis of 3D scanned foot models offers an innovative opportunity for the development of intelligent offloading device prescriptions for diabetic patients at risk of foot ulcerations. By harnessing the power of artificial intelligence and machine learning, clinicians can make data-driven, personalized recommendations to effectively offload high-risk regions on the foot and prevent the onset of diabetic foot ulcers. In this study, the performance of K-Means and GMM clustering methods in segmenting scanned foot models was evaluated. The K-Means method is generally faster and more straightforward to implement; on the other hand, the GMM approach requires more careful tuning of parameters such as the number of components and covariance structure. The results indicate that both methods are effective in delineating key anatomical structures, with the GMM demonstrating slightly higher accuracy. In practice, cluster boundaries can be translated into differential boot design parameters, such as localized thickness or stiffness adjustments, to redistribute plantar pressure or any other offloading set up.

While the ability of GMMs to better model overlapping data distributions and capture subtle variations in shape theoretically allows it to outperform K-Means for intricate geometries, the performance of both algorithms has been compared in segmenting real-world foot geometries from patients with diabetic foot ulcers. This helps provide practical insights into their effectiveness for this specific application, informing healthcare professionals’ choice on which scenario is best addressed by either algorithm.

These findings support the development of automated foot modeling and analysis tools. A proposed clinical pipeline is first to perform 3D scan acquisition, preprocessing of the mesh model and then clustering segmentation. The outcome will be used in CAD/CAM integration for the regeneration of custom boots (or orthopedic aids) for subsequent boot manufacturing. Adoption of this workflow could enable real-time offloading solutions, though barriers such as scan-to-print time and cost must be addressed in future translational studies. However, several barriers remain before routine adoption: scan-to-print time may limit applicability in urgent cases, the cost of 3D printing for advanced materials and scanning equipment remains high for some clinics, and workflow integration into multi-center healthcare settings requires validation and staff training. Addressing these challenges is essential for ensuring that clustering-driven offloading solutions become feasible, accessible, and clinically impactful.

## Data Availability

Not applicable, data in partnership with company under NDA.
